# Assessing the Feasibility of Employing a Combination of a Bacteriophage-Derived Endolysin and Spore Germinants to Treat Relapsing *Clostridioides difficile* Infection

**DOI:** 10.3390/microorganisms11071651

**Published:** 2023-06-24

**Authors:** Khalid Alyahya, Les Baillie

**Affiliations:** 1 Department of Pharmaceutics, College of Pharmacy, King Saud University, Riyadh 11451, Saudi Arabia; alyahyak@cardiff.ac.uk; 2 School of Pharmacy and Pharmaceutical Sciences, Cardiff University, Cardiff CF10 3NB, UK

**Keywords:** *Clostridium difficile*, *Clostridioides difficile*, *Clostridioides difficile* infection, CDI, endolysin, lysin, recurrent CDI, anaerobes, SEM, spore, germination

## Abstract

*Clostridioides difficile* is a Gram-positive, anaerobic, spore-forming bacillus and is a major cause of healthcare-associated infections. Whereas the vegetative form of the pathogen is susceptible to treatment with antibiotics, its ability to persist in the gut as antibiotic-resistant spores means that reinfection can occur in cases were the individual fails to re-establish a protective microflora. Bacteriophages and their lysins are currently being explored as treatment options due to their specificity, which minimizes the disruption to the other members of the gut microflora that are protective. The feasibility of employing recombinant endolysins to target the vegetative form of *C. difficile* has been demonstrated in animal models. In this study, we cloned and expressed the enzyme active domain of LysCD6356 and confirmed its ability to lyse the vegetative forms of a diverse range of clinical isolates of *C. difficile,* which included members of the hypervirulent 027 ribotype. Lytic activity was adversely affected by calcium, which is naturally found in the gut and is released from the spore upon germination. Our results suggests that a strategy in which the triggering of spore germination is separated in time from the application of the lysin could be developed as a strategy to reduce the risk of relapsing *C. difficile* infections.

## 1. Introduction

*Clostridioides difficile* (*C. difficile*) is a Gram-positive, anaerobic, spore-forming bacillus and is a major cause of healthcare-associated infection. Whereas the vegetative form of the bacillus is responsible for its pathology, primarily as a consequence of the production of two secreted toxins, TcdA and TcdB, it is the ability of the organism to form resistant spores that is responsible for the spread of the pathogen [1].

In recent years, epidemics have occurred as a consequence of the spread of spores of hypervirulent strains such as the BI/NAP1/027 ribotype [2]. In the United States, the pathogen contributes to 14,000 deaths per year [3]. In England and Wales between 2011 and 2012, *C. difficile* infection (CDI) was responsible for 15.3 deaths per million, representing a tragic loss of life [4].

Infection can manifest in varying forms, from mild diarrhoea to fatal pseudomembranous colitis. It is triggered by the administration of antibiotics, which disrupt the protective gut microflora that regulates the germination of the spore form of the pathogen. They do this by converting primary bile acids, such as taurocholate (Tc), which trigger germination into secondary bile salts that inhibit germination [5]. Asymptomatic carriage of *C. difficile* is thought to occur in 1–3% of healthy adults as a consequence of this regulatory mechanism [6].

The first-line treatment for CDI is oral vancomycin; this is followed by fidaxomicin if vancomycin proves ineffective [7]. Although these antibiotics are effective against vegetative bacilli, they have no effect on the spore form. As a consequence, residual spores re-infect the patient upon termination of antibiotic treatment in 52–88% of individuals [8]. This is thought to be due to a failure to re-establish a protective commensal flora, which is why faecal transplant, the Introduction of protective microflora from a healthy donor capable of metabolising primary bile salt, is recommended for those individuals who experience repeated episodes of infection [7].

Although antibiotics are effective, approaches that target the vegetative form of the pathogen while leaving the protective microflora unharmed are currently being explored. One such strategy is the use of *C. difficile*-specific bacteriophages, which due to their high specificity cause minimal disruption to the gut microflora and are active against antibiotic resistant isolates [9]. Whereas preliminary studies have confirmed the ability of phages to treat experimentally infected animals, progress has been hindered by the lack of lytic bacteriophages and the need to employ phage cocktails due to the development of bacteriophage resistance [10].

In an effort to address these specific issues, the use of recombinantly expressed bacteriophage-derived endolysins are also being explored [11]. These are proteins produced by bacteriophages to lyse the cell wall of the infected bacterial cell to allow the newly formed phages to escape to the outside world. Studies have shown that they are equally effective lytic agents when applied to the outer surface of vegetative bacteria as recombinant proteins. Like phages, their specificity means that they do not disrupt the gut flora and are active against antibiotic- and phage-resistant isolates.

The feasibility of employing recombinant endolysins to target the vegetative form of *C. difficile* has been demonstrated in vitro, with the first, CD27L, being reported in 2008 [11]. Subsequent work has shown that the removal of the *C. difficile* cell wall binding domain from endolysins to leave the enzyme active domain (EAD) can result in increased activity against a wider range of *C. difficile* isolates [12,13]. In an ex vivo study of infected mouse colon, the EAD of PlyCD_1–174_ significantly reduced *C. difficile* colonization and in a subsequent in vivo mouse model decreased morbidity and mortality [13]. In a further animal study, LHD, which consist of the catalytic domain of *C. difficile* phage (phiC2) endolysin (LCD) linked to the human defensin protein HD5, successfully treated infected mice [14].

Although the feasibility of treating CDI in a mouse model has been demonstrated, it should be noted that like bacteriophages, endolysins are only effective against the vegetative form of the pathogen; meaning that residual spores could potentially seed a future infection.

Interestingly, studies have shown that a combination of spore germinants and a biocidal agent can be used to clean environments contaminated with spore-forming bacteria such as *B. anthracis* and *C. difficile* [15,16,17].

In this study, we assessed the ability of the bacteriophage-derived endolysin LysCD6356 and its EAD to target clinical isolates obtained primarily in the UK. This lysin, which was isolated from φCD6356, a temperate phage of the *Siphoviridae* family, was previously shown in our hands to be more active than LysCD27 against our collection of isolates [18,19].

We also undertook a preliminary assessment of the ability of this lysin in combination with the germinants taurocholate, glycine, and calcium to target the spore form of the pathogen with a view to explore the feasibility of developing a treatment regimen that minimizes the potential for recurrent infections [20].

## 2. Materials and Methods

### 2.1. Bacterial Strains

A collection of 16 clinical isolates of *C. difficile,* representing a range of ribotypes including the principal types encountered in the UK, were kindly donated by the Anaerobe Reference Laboratory, University Hospital of Wales, Cardiff (Table 1). *E. coli* BL21 (DE3)pLysS containing the pET-19b vector was used to express endolysin (GenScript, Rijswijk, The Netherlands). Strains were stored in protect beads in Mirobank^TM^ vials (Scientific Laboratory Supplies Ltd., Nottingham, UK) at −80 °C.

### 2.2. C. difficile Spore Preparation

*C. difficile* spores were prepared as previously described [5]. Briefly, *C. difficile* R20291 was cultured on pre-reduced BHI agar and incubated under anaerobic conditions for four days. A 5 μL aliquot of cells was suspended in 1 ml sterile cold water and incubated overnight at 4 °C. The next day, the suspension was centrifuged at 5000× *g* for 5 min and the pellet was resuspended in ice-cold sterile distilled water (SDW). This was repeated five times. The suspension was then added on top of 10 mL of 50% sucrose solution and centrifuged at 3200× *g* for 20 min using a swinging bucket rotor (Heraeus Megafuge 8R centrifuge, ThermoFisher Scientific, Loughborough, UK). The sucrose layer was discarded and the pellet was washed five times with ice-cold SDW and then resuspended in SDW and stored at 4 °C.

### 2.3. Cloning of LysCD6356 and Its EAD

The gene sequence encoding LysCD6356 (NCBI reference sequence YP_004306129.1) and its enzymatically active domain were codon optimised for expression in *E. coli* and cloned into a pET-19b expression vector so that the inserted sequence was in frame with the histidine tag start codon. The plasmids were transformed into competent *E. coli* BL21 (DE3)pLysS cells (GenScript, Rijswijk, The Netherlands). For protein expression, the bacteria were cultured in LB broth containing ampicillin (100 µg/mL), with shaking (260 rpm) at 37 °C. Once the culture reached mid-log phase (OD_600_ 0.4–0.6), 1 mM IPTG was added to induce protein expression and the culture was incubated for four hours at 27 °C. Following centrifugation, the cell pellet was resuspended in lysis buffer (50 mM sodium phosphate solution, 300 mM NaCl, 10 mM imidazole, 1 mg/mL benzonase nuclease and 1 mg/mL lysozyme; pH 8) and incubated at room temperature for 30 min. The bacterial suspension was then sonicated and centrifuged at 14,000× *g* at 4 °C for 30 min. The histidine tagged recombinant protein was purified using a Ni-NTA affinity column as per the Qiagen instructions and was eluted using a range of imidazole concentrations (50–250 mM) to identify the most appropriate concentration. The protein purity was analysed by SDS-PAGE and protein concentration was determined using the BCA assay (Fisher Scientific, Leicestershire, UK). The relative purity was determined using Bio-Rad software (ChemiDoc^TM^ XRS+ 6.0).

### 2.4. Assessing the Lytic Activity of EAD

Lytic activity against vegetative *C. difficile* isolates was determined using a turbidity reduction assay [11,19]. Isolates were cultured in 45 mL of pre-reduced BHI broth at 37 °C under anaerobic conditions to the mid-logarithmic phase (OD_600_ 0.4–0.6). Following centrifugation (5000× *g* for 5 min), the pellets were resuspended in phosphate-buffered saline (pH 7.4) and adjusted to OD_600_ 0.8–1.2. A 300 μL aliquot of cell suspension was mixed with recombinant endolysin in PBS in a Costar 96 flat transparent well plate. As a negative control, the endolysin was replaced by PBS. The plates were incubated under aerobic conditions at 37 °C and changes in OD_600_ over time were determined using a TECAN Infinite F200 Pro plate reader at fixed time points. To normalize the results, the initial value (OD_600_ a time 0) was adjusted to an OD reading of 1.0. Experiments were performed in triplicate for each test condition.

### 2.5. The Impact of pH on EAD Activity

Vegetative *C. difficile* cells grown to mid-log phase (OD_600_ 0.4–0.6) were centrifuged at 5000× *g* for 5 min and resuspended in PBS as pH levels ranging from 4 to 9. 60 µg/mL EAD was added to 300 μL of the various suspensions in a Costar 96 flat transparent well plate. Changes in the OD_600_ over time were determined as described above.

### 2.6. The Impact of Calcium on EAD Activity

Vegetative *C. difficile* cells grown to mid-log phase (OD_600_ 0.4–0.6) were centrifuged at 5000× *g* for 5 min and resuspended in different concentrations of CaCl_2_ in SDW (0, 12.5, 25, 50 and 100 mM). A total of 60 µg/mL the EAD was added to 300 μL of the various suspensions in a Costar 96 flat transparent well plate. Changes in the OD_600_ over time were determined as described above.

### 2.7. The Contribution of Divalent Metal Cations to EAD Activity

The EAD (60 µg/mL) was incubated with 250 mM ethylenediaminetetraacetic acid (EDTA) at 37 °C for 30 min [26]. EDTA was then removed using a 10,000 MWCO Pierce^TM^ protein concentrator, and the treated protein resuspended in sterile deionized water (DIW). The lytic activity of the EDTA-treated EAD was determined as described above and compared with that of untreated EAD.

To identify the cations involved in EAD activity, 60 µg/mL of EDTA-treated EAD was mixed with 1 mM Mg^2+^, Mn^2+^ or Zn^2+^ and then added to 300 μL of resuspended vegetative *C. difficile* R20291 [26]. The lytic activity was determined as described above and compared with that of untreated EAD.

### 2.8. Impact of Spore Germinants and Calcium Chloride on the Lytic Activity of the EAD

The impact of the spore germinants, sodium taurocholate (Tc) and glycine, in the presence and absence of CaCl_2,_ on the ability of the EAD to lyse vegetative cells of *C. difficile* R20291 was determined. Mid-log-phase cells (OD_600_ 0.4–0.6) were centrifuged at 5000× *g* for 5 min and resuspended in 300μL of the following, DIW, DIW + 0.1% Tc + 50 mM glycine; DIW + 12.5 mM CaCl_2_; DIW + 0.1% Tc + 50 mM glycine + 12.5 mM CaCl_2_; DIW + 12.5 mM CaCl_2_ + 60 µg/mL EAD; DIW + 60 µg/mL EAD; DIW + 0.1% Tc + 50 mM glycine + 12 mM CaCl_2_ + 60 µg/mL EAD [13]. Plates were incubated under aerobic condition at 37 °C and changes in OD_600_ over time were determined using a TECAN Infinite F200 Pro plate reader at fixed time points. To normalize results, the initial value (OD_600_ at time 0) was adjusted to an OD reading of 1.0. Experiments were performed in triplicate for each test condition.

### 2.9. C. difficile Spore Germination

Spore germination was assessed in the presence of 0.1% sodium taurocholate and 50 mM glycine with and without 12.5 mM calcium chloride. Purified *C. difficile* spores were adjusted to start at ~1 OD_600_. Each germination experiment was performed in a Costar 96-well flat transparent microtitre plate. Germination was monitored by measuring the change in the OD_600_ reading at 37 °C using an TECAN Infinite F200 Pro plate reader. The experiment was performed in triplicate.

### 2.10. Assessing the Sensitivity of Newly Germinated Spores to the EAD

#### 2.10.1. Simultaneous Exposure to Spore Germinants and the EAD

*C. difficile* R20291 spores at a starting OD_600_ of ~1.0 were induced to germinate by the addition of the following germinants: 0.1% Tc and 50 mM glycine with and without 12.5 mM CaCl_2_ in SDW. Simultaneously, 60 µg/mL of the EAD was added to each test group. R20291 spores were re-suspended in SDW without germinants as a negative control.

#### 2.10.2. Sequential Exposure to Spore Germinants and the EAD

*C. difficile* spores at a starting OD_600_ of ~0.1 were incubated anaerobically with the germinants 0.1% Tc and 50 mM glycine for 150 min in SDW or BHI at 37 °C. The treated suspensions were then centrifuged and resuspended in PBS to an OD_600_ of 1.0. A 300 μL sample of each test group was loaded in a Costar 96 flat transparent well plate and 60 µg/mL EAD was added. Positive and negative controls were included by suspending vegetative *C. difficile* R20291 in PBS alone or in the presence of 60 µg/mL EAD.

### 2.11. Phase-Contrast Microscopy

Imaging using phase-contrast microscopy was performed as previously described [27]. Briefly, 5 μL of the bacterial samples was mounted using 80% glycerol solution on a microscopic slide and a cover slip carefully placed over the sample. Cells were then observed under Leica DMIRB Inverted Leica Modulation Contrast Microscope. Images were analysed and a scale bar was applied using ImageJ 1.53k software.

### 2.12. Scanning Electron Microscopy

A *C. difficile* suspension was mixed with a fixative solution (2% glutaraldehyde in 0.1 M cacodylate buffer, pH 7.4) (Generon, Slough, UK) at a 1:9 ratio and incubated for 2 h at room temperature. Following fixation, the cells were resuspended in 0.1 M sodium cacodylate buffer (pH 7.4) and incubated at room temperature for 3 min. After this, the cells were resuspended in increasing concentrations of ethanol (50%, 70%, 80%, 95% and 100%). The treated cells were recovered by filtration using a 0.2 μm pore polycarbonate filter membrane. Following the removal of the solution, the membrane was cut into 1 cm squares that were mounted on stainless steel studs. The membrane was sputter coated with 7 nm of an 80/20 Au/Pd mixture and transferred to the electron microscope vacuum chamber. The cells were then visualized using a 5 kV electron beam.

### 2.13. Statistical Analysis

All statistical analysis were carried using GraphPad Prism 9 software. For analysing differences between two groups, a t-test analysis was used. One-way ANOVA was used for differences among more than two groups.

## 3. Results

### 3.1. Production of Full-Length Recombinant Lysin of CD6356 and Its EAD Fragment from an E. coli Expression Host

The recombinant forms of the full-length CD6356 lysin and its EAD fragment were expressed from *E. coli* (Figures S1 and S2). Recombinant protein yields of the full-length CD6356 and its EAD were 350 µg/L and 300 µg/L, respectively. The purity of the full-length monomeric CD6356 was 82.2%, whereas the EAD made up 73.8% of the protein in the sample.

### 3.2. Enzymic Activity of LysCD6356 and Its EAD Fragment against Vegetative Isolates of C. difficile

We first confirmed the biological activity of the recombinantly expressed proteins against R20291, a hypervirulent 027 ribotype strain isolated from a major outbreak in the UK that has been characterized across a number of studies [28,29,30,31] (Figure S3).

**Figure 1:**
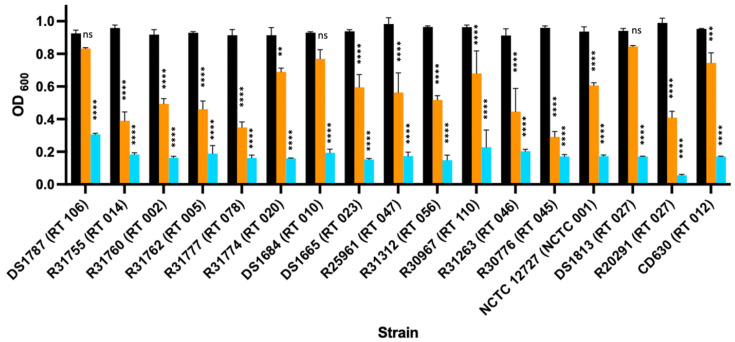
Lytic activity of LysCD6356 and its EAD fragment against a collection of clinical isolates of *C. difficile.* Vegetative forms of the test bacteria suspended in PBS to an OD600 of 1.0 were incubated with either full-length lysin (orange bar) or EAD (blue bar) at a concentration of 60 µg/mL for 60 min at 37 °C, at which time the OD_600_ was recorded. Suspended bacteria without treatment were used as controls (black bars). *p* value < 0.01 (**), *p* < 0.001 (***), *p* < 0.0001 (****).

We next determined their activity against a diverse panel of clinical isolates of over a 60 min time period. The lytic activity of the full-length lysin varied depending on the test strain (Figure 1).

The EAD was more active than the full-length lysin. This finding is in keeping with other studies that have also shown that the removal of the cell binding domain increases the lytic activity and the range of isolates that can be lysed [12,13].

### 3.3. Effect of pH on Lytic Activity

We next determined the ability of the EAD fragment to lyse vegetative bacteria across a range of pH values, as the pH is known to affect the activity of *C. difficile*-specific phage-derived recombinant lysins [12,13,14,19,32]. Similar to the full-length lysin, the EAD fragment retained activity against the vegetative form of R20291 across a pH range of 4–9 (Figure S4).

### 3.4. The Role of Divalent Cations in the Enzymic Activity of the EAD

**Figure 2:**
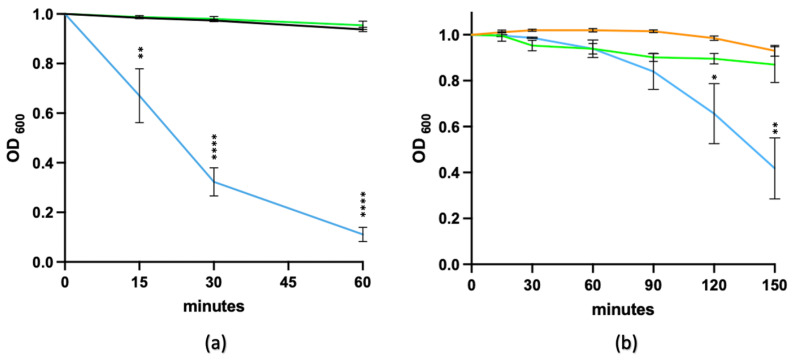
The contribution of divalent cations to the enzymic activity of LysCD6356 EAD. (**a**) Lytic activity of the EAD treated with a chelating agent against vegetative R20291. A total of 60 µg/mL EAD was incubated with 250 mM EDTA for 30 min. Vegetative R20291 resuspended in DIW (black line), vegetative R20291 resuspended in DIW with EAD following EDTA treatment (green line) or vegetative R20291 resuspended in DIW with EAD (blue line) are shown. (**b**) Restoration of the lytic activity of EDTA-treated EAD by Zn^2+^ ions. The ability of Zn^2+^ to restore the lytic activity of EDTA-treated recombinant EAD following the addition of 1 mM Zn^2+^ solution was assessed against vegetative R20291 (blue line). As a control, vegetative R20291 was suspended with EDTA (green line) and with Zn^2+^ (orange line). Experiments were performed in triplicate. Changes in OD_600_ were normalized to the initial value. Statistical analysis was performed on treated and untreated groups. *p* value = < 0.05 (*), *p* < 0.01 (**), *p* < 0.0001 (****).

The lytic activity of the enzyme active domain of the *C. difficile*-derived lysin, CD27L_1–179_, which, like LysCD6356, is an N-acetylmuramyl-L-alanine amidase, depends on the presence of zinc in its catalytic centre [12]. To determine if this was also the case for the EAD of LysCD6356, the recombinant protein was incubated in the presence of the chelating agent EDTA for 30 min prior to testing for lytic activity against R20291. As can be seen in Figure 2a, the lytic activity of the EAD was lost, indicating the importance of cations in the functionality of the lysin. The addition of Zn^2+^ ions to EDTA-treated EAD restored the lytic activity, with the level of regained activity increasing with time (Figure 2b), which was not the case with addition of Mn^2+^ and Mg^2+^ (Figure S5).

### 3.5. The Impact of Calcium on the Enzymic Activity of the EAD of LysCD6356

**Figure 3:**
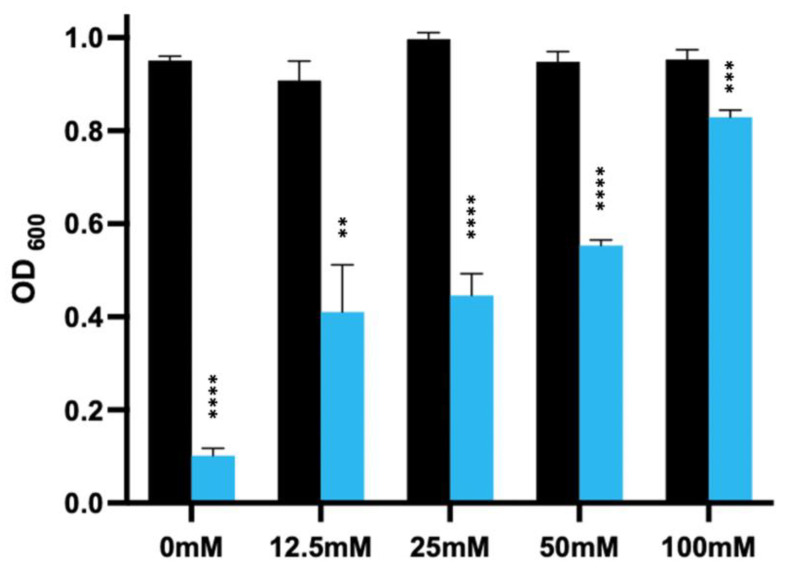
The lytic activity of 60 µg/mL EAD against the vegetative form of *C. difficile* R20291 in the presence of increasing concentrations of calcium chloride following a 60 min incubation. Vegetative *C. difficile* strain R20291 was used to assess the activity of 60 µg/mL of the EAD of LysCD6356 in the presence of different concentrations of calcium chloride (pH 7 and 37 °C). The effect on lysis was determined by measuring the change in the OD_600_ in the presence (blue) or absence (black) of calcium. The experiment was performed in triplicate. *p* value < 0.01 (**), *p* < 0.001 (***), *p* < 0.0001 (****).

It has been reported that calcium can adversely affect the lytic activity of bacteriophage-derived lysins [13]. This is important as calcium is present in the gut and *C. difficile* spores release a store of calcium as part of the germination process [33]. To determine if this was the case for the EAD of LysCD6356, we analysed the effect of increasing concentrations of calcium chloride on the lytic activity of EAD against the vegetative form of R20291 following a 60 min incubation at 37 °C [13]. As can be seen from Figure 3, the presence of calcium significantly reduced the lytic activity of the enzyme and this inhibition increase as the concentration of calcium chloride increased.

Interestingly, we still saw activity at concentrations above 25 mM, which are likely to be above the levels of the local calcium concentration present in the gut.

### 3.6. Effect of Spore Germinants and Calcium on the Activity of the EAD against Vegetative R20291

**Figure 4:**
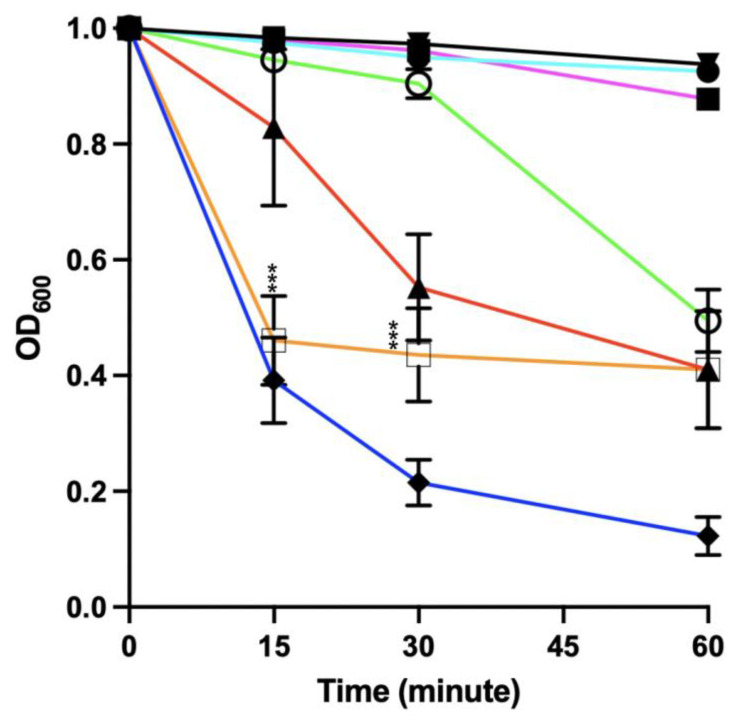
The impact of germinants and calcium on the lytic activity of the EAD of LysCD6356 against vegetative R20291. The *C. difficile* strain R20291 was grown to mid-log phase and resuspended in DIW (black line), DIW with 12.5 mM CaCl_2_ (light blue line), DIW with germinants (0.1%Tc and 50 mM glycine) (purple line), DIW with germinants (0.1%Tc and 50 mM glycine) and 12.5 mM calcium (green line), DIW with 12.5 mM CaCl_2_ and EAD (red line), DIW with germinants, calcium and EAD (orange line) or DIW with EAD (dark blue line). For all experimental groups, changes in OD_600_ were normalized to the initial value and the experiment was performed in triplicate at 37 °C. Statistical analysis was performed between the combination of germinants and CaCl_2_ with and without the EAD against vegetative R20291. *p* value < 0.001 (***).

We next determined the impact of the spore germinant sodium taurocholate (Tc) and the co-germinant glycine on the ability of the EAD to lyse vegetative *C. difficile*. The presence of these germinants had no adverse effect on the activity of the lysin (Figure S6). We next determined if the presence of calcium at 12.5 mM in addition to the germinants had any impact on the lytic activity. This level of calcium was selected to reflect the level of calcium reported to be present in the mouse ileum [34]. As can be seen from Figure 4, the presence of 12.5 mM calcium chloride reduced the lytic activity of the EAD alone and in the presence of the germinants.

We also saw a reduction in the optical density of vegetative bacteria in the absence of the EAD but in the presence of Tc, glycine and calcium, an effect that was not seen with Tc and glycine or calcium chloride alone (Figure 4). These results suggest the combination of calcium and germinants may possess some form of direct activity in the absence of the EAD. Similar results were observed when higher concentrations of calcium chloride (25 and 50 mM) were combined with 0.1% Tc and 50 mM glycine.

### 3.7. Characterisation of Bacterial Structure Using Scanning Electron Microscopy

**Figure 5:**
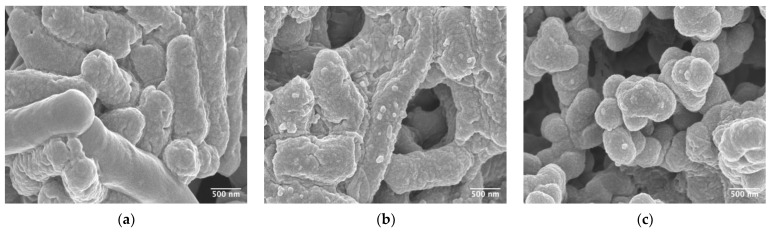
Scanning electron microscope images of *C. difficile* R20291 subjected to the EAD of LysCD6356. Vegetative *C. difficile* strain R20291 was incubated in BHI broth to mid-log phase, after which cells were resuspended in SDW. (**a**) No EAD, (**b**) 60 µg/mL EAD for 15 min and (**c**) 60 µg/mL EAD for 30 min. Images are at 70,000× magnification.

To visualize the impact of the various treatments on the structure of the vegetative bacterium, we examined treated samples using scanning electron microscopy. As can be seen in Figure 5, exposure of the vegetative bacteria to the lysin caused disruption of the cells surface that increased with time.

In the presence of taurocholate and glycine, this disruption was still evident (Figure S7a). Examination of the images of vegetative bacteria exposed to germinants and calcium in the absence of the EAD revealed no obvious evidence of cellular disruption, although there was a marked reduction in the length of the bacteria from 4.46 μm (±1.05) to 1.73 μm (±0.28) when calcium was added to the germinant mixture (Figures S8 and S9). This reduction in size would explain the reduction in the optical density observed in Figure 4.

### 3.8. Spore Germination

We next determined the ability of our germination mixture in either 20 mL DIW or BHI to trigger the germination of R20291 spores. The impact of adding 12.5 mM of calcium chloride to the germination mixture was also determined. The reduction in optical density to an OD_600_ of 0.55 is indicative of complete spore germination and was confirmed using phase-contrast microscopy (Figure S10) [11].

**Figure 6:**
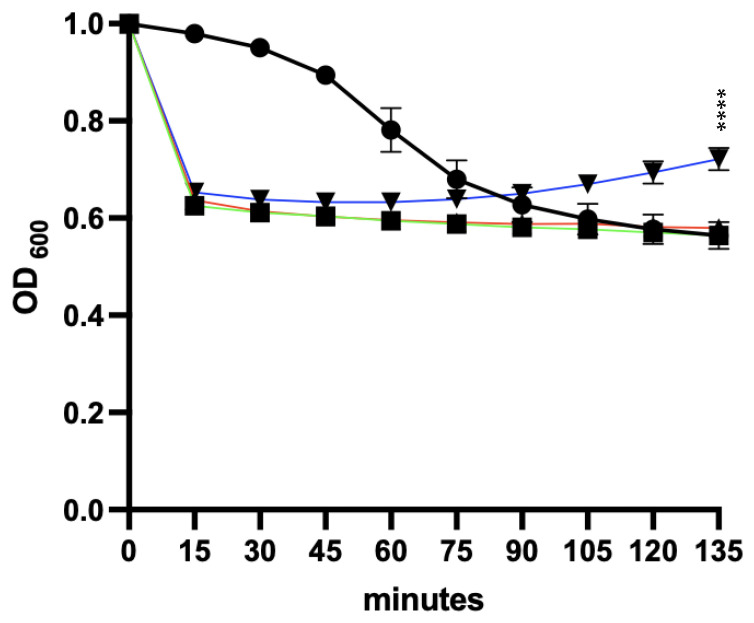
Germination of *C. difficile* R20291 spores following exposure to a mixture of germinants. R20291 spores were incubated in the presence of different germination mixtures for 150 min at 37 °C and the change in OD_600_ was determined. The following germination mixtures were used: 0.1% Tc and 50 mM glycine in DIW (black line), 0.1% Tc, 50 mM glycine and 12.5 mM calcium chloride in DIW (green line), 0.1% Tc and 50 mM glycine in BHI (red line) or 0.1% Tc, 50 mM glycine and 12.5 mM calcium chloride in BHI (blue line). The experiment was performed in triplicate. Statistical analysis was performed between the combination of R20291 spores, germinants and CaCl_2_ in BHI or DIW and between the combination of R20291 spores and germinants in BHI with or without CaCl_2_. *p* value < 0.0001 (****).

Although we saw a reduction in the optical density indicative of germination in the presence of 0.1% Tc and 50 mM glycine in DIW, the rate of germination was considerably faster in the remaining germination mixtures, with the majority of spores germinating in approximately 15 min (Figure 6). These results suggest that the presence of calcium chloride had enhanced the rate of germination of the R20291 spores and supported outgrowth in the presence of BHI.

### 3.9. Sensitivity of Newly Germinated C. difficile to Lysis by the EAD

We next determined the ability of the EAD to lyse newly germinated spores of R20291. We compared the effect of delivering the EAD at the same time as the germination mixture to that of delivering them sequentially, i.e., germinants followed by a washing step and then the EAD.

#### 3.9.1. Co-Delivery of Germinants and the EAD

**Figure 7:**
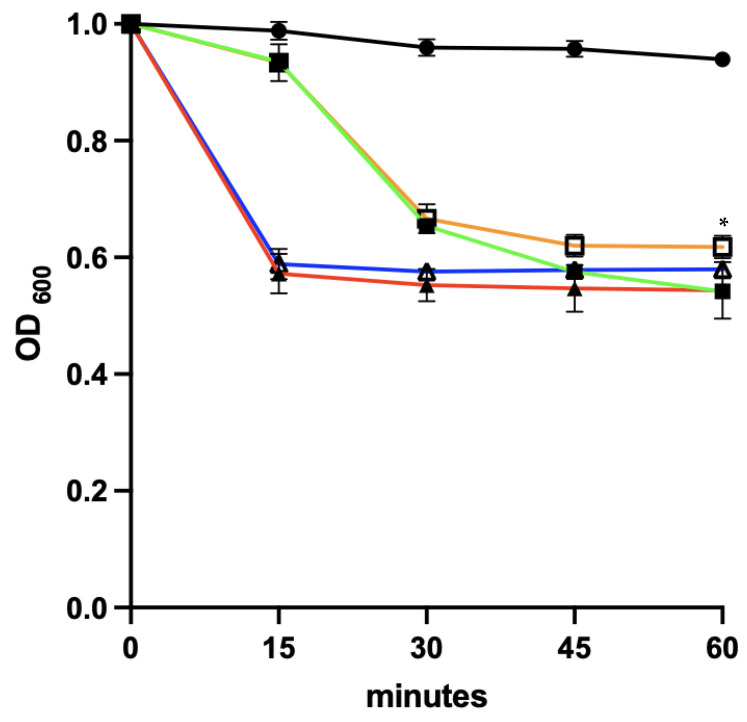
The effect of treating *C. difficile* R20291 spores with germinants, calcium and EAD at the same time. R20291 spores was treated with a germinant mixture comprising 0.1% Tc and 50 mM glycine (green line), germinants with 60 µg/mL recombinant EAD (orange line), germinants with 12.5 mM CaCl_2_ (red line) or germinants with 12.5 mM CaCl_2_ and 60 µg/mL recombinant EAD (blue line) at 37 °C. R20291 spores were suspended in DIW (black solid line with circles) as a control. The change in the OD_600_ was monitored throughout the experiment. The experiment was performed in triplicate. Statistical analysis was performed between R20291 spores exposed to germinants with and without 60 µg/mL EAD. *p* value ≤  0.05 (*).

As can be seen from Figure 7, spore germination was faster in the presence of additional calcium, although both germination conditions achieved a similar end point at 60 min. We saw no evidence of lysis in the presence of the EAD. This could be due to the release of calcium trapped within the spore or due to the fact that newly germinated spores are relatively resistant to EAD-mediated lysis [35].

#### 3.9.2. Germinants Followed by the EAD

**Figure 8:**
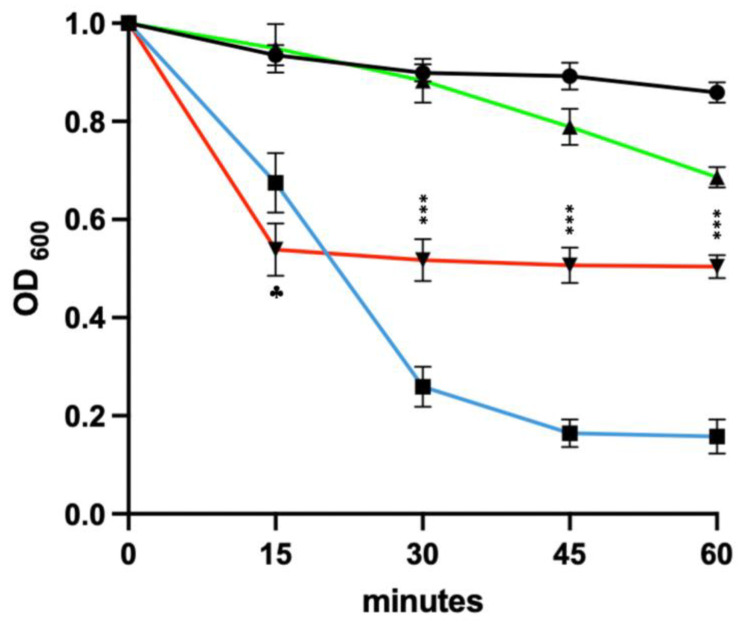
The impact of treating newly germinated spores of R20291 with recombinant EAD following a washing step. Spores of R20291 were incubated with germinants and pre-reduced BHI for 150 min. Following germination, the vegetative bacteria were washed with PBS and resuspended to an OD_600_ of 0.8 to 1.2 in PBS (green line) or PBS with 60 µg/mL EAD (red line). Vegetative *C. difficile* obtained from a BHI culture and resuspended in PBS (black line) or PBS with 60 µg/mL EAD (blue line) were used as controls. The experiment was performed in triplicate and readings were normalized to the initial value. Statistical analysis was performed on germinated spores with and without exposure to 60 µg/mL EAD. ♣ refers to a significant (***) reduction in germinated spores 15 min after the addition of 60 µg/mL EAD. *p* value < 0.001 (***).

Finally, we determined the impact of delaying the addition of the EAD to the newly germinated spores on the subsequent lysis of the vegetative organisms. Following germination, the vegetative cells were washed with PBS and resuspended to an OD_600_ of 1 in the presence of the EAD (Figure S11). As can be seen in Figure 8, the germinant-treated spore sample showed a progressive reduction in the optical density, presumably due to the germination of residual spores. In the presence of the EAD, the germinant-treated culture showed a significant reduction in optical density that was suggestive of lysin-mediated disruption.

## 4. Discussion

We assessed the ability of the bacteriophage-derived endolysin LysCD6356 and its EAD to target clinical isolates of *C. difficile* obtained primarily in the UK. Using an endolysin expression protocol developed in an earlier study, we attempted to optimise the expression of LysCD6356 and its EAD from an *E. coli* host [19]. Surprisingly, our expression levels were not as high as those reported in previous studies including our own [19,36]. The lower levels of expression could reflect variations in the efficiency of codon optimisation or the use of different strains of *E. coli* to express the proteins [36,37].

Consistent with prior research findings, the EAD was more active than full-length LysCD6356 against our collection of clinical isolates [12,13]. The EAD shares 77.5% and 34.7% amino acid sequence identity with PlyCD and CD27L, respectively, suggesting that it may be a zinc-dependent N-acetylmuramoyl-L-alanine amidase [36]. This hypothesis was confirmed by the ability of zinc to restore the biological activity of the EDTA-treated protein. The EAD also demonstrated a similar spectrum of enzymic activity to that of CD27L across a wide range of pH values [11].

In addition to determining the contribution of zinc to biological activity, we also examined the impact of calcium, as this compound occurs naturally in the gut and has been reported to inhibit the lytic activity of PlyCD [13]. We found that calcium also inhibited the lytic activity of the EAD in a concentration-dependent manner. Interestingly, the enzyme retained significant lytic activity at concentrations of calcium in excess of the levels reported to occur in the gut (15 mM), suggesting that this inhibitory effect could be overcome in situ by increasing the local concentration of the lysin.

Given that our ultimate aim was to eliminate the spore form of the pathogen from the gut, we next determined the ability of the EAD to lyse the vegetative form of *C. difficile* in the presence of spore germinants. Although sodium taurocholate and glycine had no impact on lytic activity, the addition of calcium chloride inhibited lysis in a concentration-dependent manner.

Interestingly, the combination of sodium taurocholate, glycine, and calcium in the absence of the EAD significantly reduced the length of the vegetative bacterium. During the course of infection, vegetative *C. difficile* encounters multiple stresses in the gastrointestinal tract, including exposure to bile salts and high osmolarity [38,39]. Bile salts have detergent properties that can modify the bacterial cell wall in manner that reduces the organism’s osmotic tolerance [40]. It may be the case that the reduction in size was due to the combination of bile-salt-induced cell wall disruption and osmosis-driven leakage of intracellular contents due to the relativity high concentration of extracellular calcium.

Following the confirmation of the EAD’s capability to lyse vegetative bacteria in the presence of germinants, the efficacy of the lysin in targeting the spore form of the pathogen was subsequently assessed.

The simultaneous delivery of germinants and lysin was not as effective as delivering them separately. It is possible that the rapid release of calcium held within the cortex of the spore upon the triggering of germination when combined with the calcium already present in the germination mixture was sufficient to inhibit lytic activity. Another possibility is that the newly germinated organisms are relatively resistant to EAD-mediated lysis. Mehta and colleagues observed that spores of the Sterne strain of *B. anthracis* required at least 2 h after the exposure to germinants before the actively growing organism developed sensitivity to lysin-mediated disruption [35].

Delivery of intact proteins such as lysins to the lower GI tract is a particular challenge due to the harsh pH encountered in the stomach and the presence of degradative proteases. One option could be to express the lysin in situ from a bacterial host such as *Lactococcus lactis*, which is a food-grade probiotic organism that has been genetically engineered to express a recombinant protein. Previous studies have confirmed the feasibility of using this approach to produce therapeutic proteins in the GI tract [41]. Mayer and colleagues reported that biologically active *C. difficile* ΦCD27 endolysin could also be expressed from *L. lactis,* raising the possibility that this could be a viable delivery platform [11].

Thus, one could envision a scenario in which patients who had experienced an episode of CDI were given a lysin-expressing strain of *L. lactis* to colonise the gut followed sometime later by a mixture of germinants and calcium. The continued expression of the lysin in the gut could potentially overcome temporary germination-mediated inhibition of lysin activity.

In conclusion, we have cloned and expressed the EAD of LysCD6356 and confirmed its ability to lyse the vegetative form of a diverse range of clinical isolates of *C. difficile,* which included members of the hypervirulent 027 ribotype. Our results suggest a strategy in which spore germination followed several hours later by exposure to lysin could potentially be developed as a strategy to reduce the risk of relapsing CDI.

## Figures and Tables

**Table 1:** *C. difficile* isolates used in this study and their clinical relevance.

Strains	Ribotypes	Clinical Relevance	References
NCTC 12727	001	Increased resistance to vancomycin	[21]
R31760	002	The most common ribotype in England	[22]
R31762	005	Third most common ribotype in England	[22]
DS1684	010		
CD630	012	Came from an outbreak in Zurich, Switzerland; first strain to be genome sequence	[23]
R31755	014	Fourth most common ribotype in England	[22]
R31774	020	Fifth most prevalent ribotype in England	[22]
DS1665	023	The 10^th^ most common ribotype in England	[22]
DS1813/ R20291	027	Hypervirulent, most common ribotype in Texas, USA. R20291 isolated in 2006 from an outbreak in Stoke Mandeville Hospital, England	[24]
R30776	045		
R31263	046		
R25961	047		
R31312	056		
R31777	078	Sixth most common ribotype in England	[22]
DS1787	106	The most common ribotype in the USA	[25]
R30967	110		

## Data Availability

Data presented in this study are available on request from the corresponding author.

## Supplementary Materials

The following supporting information can be downloaded at: https://www.mdpi.com/article/10.3390/microorganisms11071651/s1. Figure S1. SDS-PAGE and Western blot analysis of different fractions of recombinant LysCD6356 following elution through an Ni-NTA column and the removal of Imidazole.; Figure S2. SDS-PAGE and Western blot analysis of different fractions of recombinant EAD of LysCD6356 following elution through an Ni-NTA column and the removal of Imidazole.; Figure S3. The lytic activity of 7.5 and 60 μg/ml of LysCD6356 and its EAD against *C. difficile* strain R20291.; Figure S4. Impact of pH on the lytic activity of 60 µg/ml EAD of LysCD6356 at 60 minutes.; Figure S5. The impact of divalent cations to the enzymic activity of LysCD6356 EAD.; Figure S6. The role of germinants on the activity of EAD; Figure S7. Scanning electron microscope images of *C. difficile* R20291 exposed to EAD of LysCD6356 and germinants in presence and absence of calcium.; Figure S8. Scanning electron microscope images of *C. difficile* R20291 exposed to germinants in presence and absence of calcium.; Figure S9. Size comparison between vegetative and germinants with and without calcium.; Figure S10. Phase contrast images of the germination of *C. difficile* spores.; Figure S11. Phase contrast imaging of *C. difficile* spores R20291 following 150 minutes of germination.
