# Supplementary material for: Assessing the Feasibility of Employing a Combination of a Bacteriophage-Derived Endolysin and Spore Germinants to Treat Relapsing Clostridioides difficile Infection
Source: Microorganisms. 2023 Jun 24;11(7):1651. doi: 10.3390/microorganisms11071651 (PMC10384137; doi:10.3390/microorganisms11071651)
Supplement: Supplementary file 1 [file microorganisms-11-01651-s001.zip › microorganisms-2317810-supplementary.pdf]

microorganisms

MDPI

## Supplement

*1. Production of full-length recombinant lysin of CD6356 and its EAD fragment from an E. coli expression host*

We expressed and purified recombinant forms of the full-length CD6356 lysin and its EAD fragment. The presence of each protein was confirmed by Western blotting using a monoclonal antibody specific to the histidine tag located at the N terminus of the recombinant protein and by screening for enzymic activity. As can be seen from figure S1, the major band corresponding to the predicted size of LysCD6356 (32.67 kDa) gave a strong signal with the antibody. Minor bands were also recognized in the concentrated sample, suggesting that breakdown products and a multimeric form of LysCD6356 may be present. Using Bio-Rad software, we estimated the proportion of recombinant LysCD6356 in the purified sample to be 88.1%, of which 82.2% was monomeric.

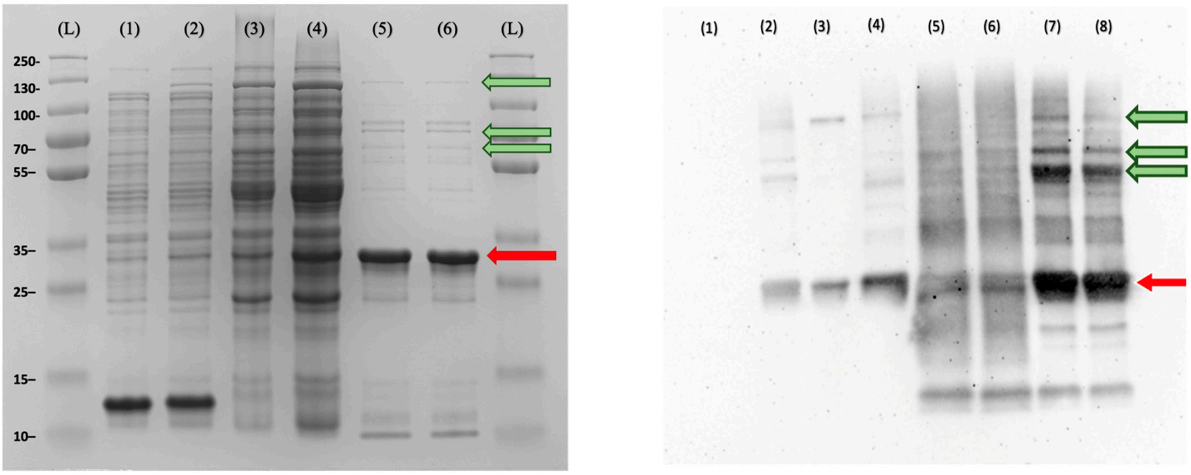


(a) (b)

**Figure S1.** SDS-PAGE and Western blot analysis of different fractions of recombinant LysCD6356 following elution through a Ni-NTA column and the removal of imidazole. (a) Coomassie Blue staining of an SDS-PAGE gel. (1) Pre-induction fraction, (2) post-induction fraction, (3) flow through fraction, (4) wash fraction, (5) and (6) elution fraction following removal of imidazole and (L) molecular weight ladder (PageRuler™ Plus Prestained Protein Ladder). (b) A Western blot analysis of recombinant LysCD6356. (1) Pre-induction fraction, (2) post-induction fraction, (3) flow through fraction, (4) wash fraction, (5) and (6) elution fraction before the removal of imidazole (250mM) and the concentrated fraction, (7) and (8) elution fraction following the removal of imidazole. The red arrows represent the predicted location of LysCD6356 (~32.67kDa), whereas green arrows represent multimeric forms of LysCD6356.

The same approach was employed to express and characterise the recombinant EAD fragment of the lysin.

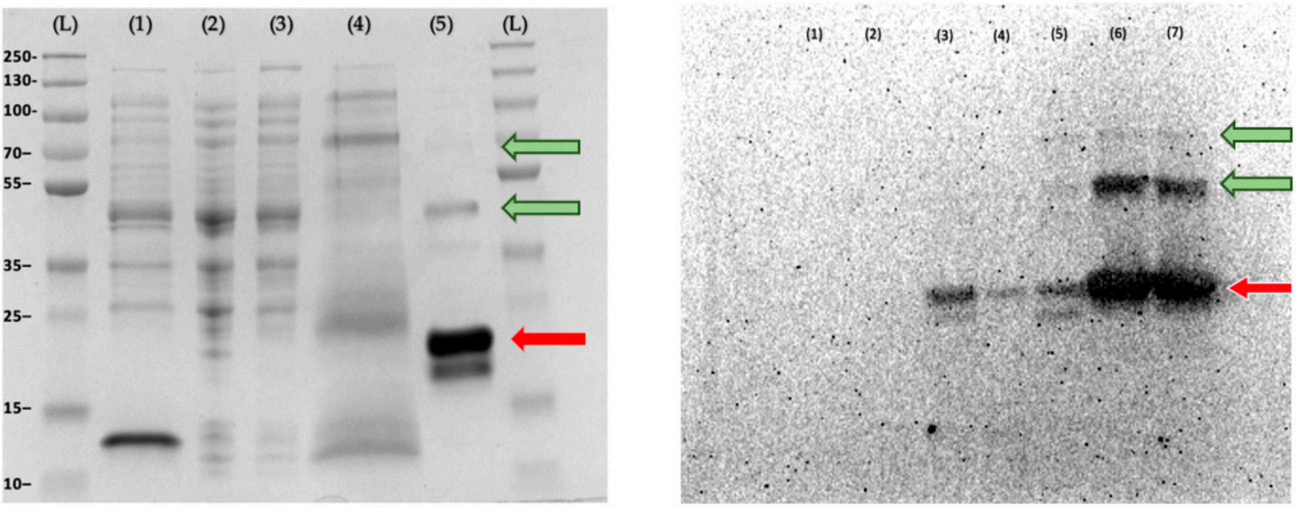


(a) (b)

**Figure S2.** SDS-PAGE and Western blot analysis of different fractions of the recombinant EAD of LysCD6356 following elution through a Ni-NTA column and the removal of imidazole. (a) Protein band detection using Coomassie Blue staining on SDS-PAGE. (1) Pre-induction fraction, (2) post-induction fraction, (3) flow through fraction, (4) wash fraction, (5) elution fraction after the removal of imidazole (250mM) and (L) molecular weight ladder (PageRuler™ Plus Prestained Protein Ladder). (b) A Western blot analysis of the recombinant EAD of LysCD6356. (1) Pre-induction fraction, (2) blank, (3) post-induction fraction, (4) flow through fraction, (5) wash fraction, (6) and (7) elution fraction after the removal of imidazole (250mM). The red arrow represents the predicted location of the EAD of LysCD6356 band (22.12 kDa), whereas green arrows represent multimeric forms of the EAD.

As can be seen from figure S2, the major band corresponding to the predicted size of the EAD (22.12 kDa) gave a strong signal with the antibody. The next strongest band running below the main band was found to the oxidized form of the recombinant lysin. Other minor bands recognized by Western blot in the concentrated sample suggest the presence of multimeric and breakdown forms of the EAD. Using Bio-Rad software, we estimated the proportion of the recombinant EAD in the purified sample to be 86.1%, of which 73.8% was monomeric.

## *2. Activity of Endolysins Against C. difficile Strain R20291*

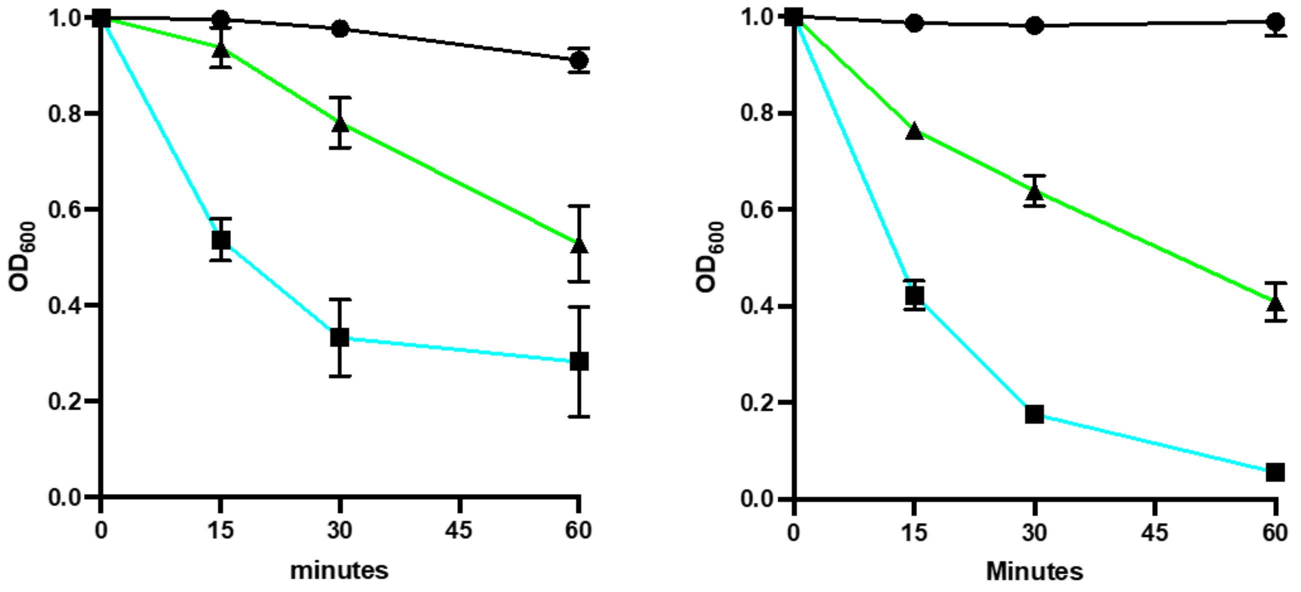


(a) (b)

**Figure S3.** The lytic activity of 7.5 and 60 µg/ml of LysCD6356 and its EAD against *C. difficile* strain R20291**.** The *C. difficile* strain was cultured in BHI broth to mid-log phase and resuspended in PBS (pH 7.4) (black line). (a) 7.5 µg/ml and (b) 60 µg/ml of LysCD6356 (green line) and its EAD (blue line) were added to the suspension and incubated at 37° C. The changes in the $OD_{600}$ were recorded using a TECAN Infinite F200 Pro plate reader. The experiment was performed in triplicate.

*3. Effect of pH on Lytic Activity.*

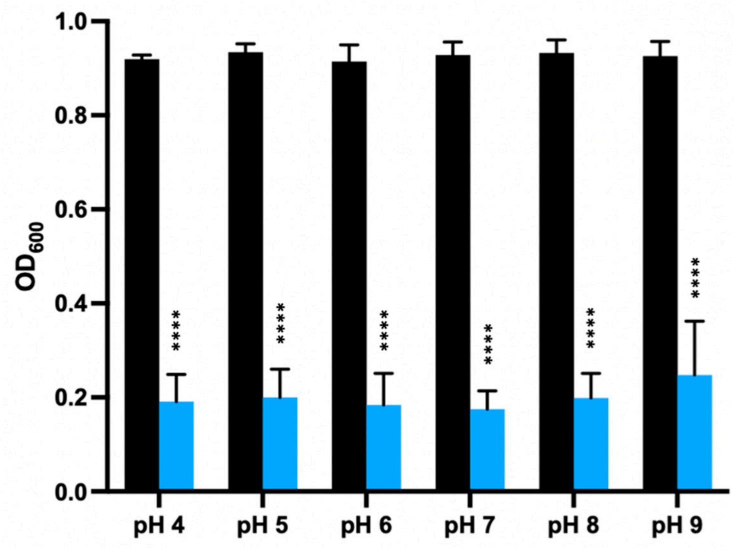


**Figure S4.** Impact of pH on the lytic activity of 60 µg/ml of the EAD of LysCD6356 at 60 minutes. *C. difficile* strain R20291 was grown to mid-log phase and resuspended in PBS with different pH levels (pH 4 to 9) (black bar). A total of 60 µg /ml of the EAD was added to each pH level (blue bar). The experiment was performed in triplicate. *p* value < 0.0001 (****).

*4. The Role of Divalent Cations in the Enzymic Activity of the EAD.*

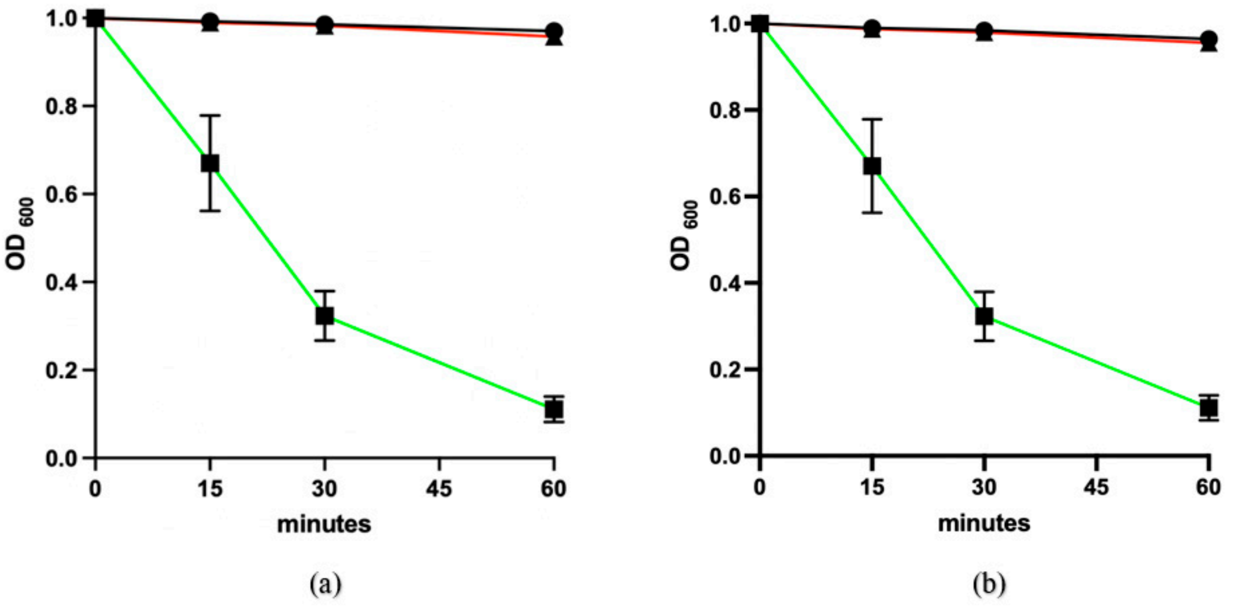


**Figure S5.** The impact of divalent cations on the enzymic activity of the EAD of LysCD6356. The lytic activity of EAD treated with a chelating agent against vegetative R20291. Restoration of the lytic activity of EDTA-treated EAD by $Mg^{2+}$(a) or $Mn^{2+}$(b) ions. The ability of divalent cations to restore the lytic activity of EDTA-treated recombinant EAD following the addition of 1mM of either $Mg^{2+}$(a) or $Mn^{2+}$ (b) solutions was assessed against vegetative R20291 (red line). As a control, vegetative R20291 was suspended with untreated 60μg/ml EAD (green line) and with either $Mg^{2+}$(a) or $Mn^{2+}$ (b) solutions (black line). Experiments were performed in triplicate. Changes in OD600 were normalized to the initial value.

*5. The Role of Germinants on the activity of the EAD.*

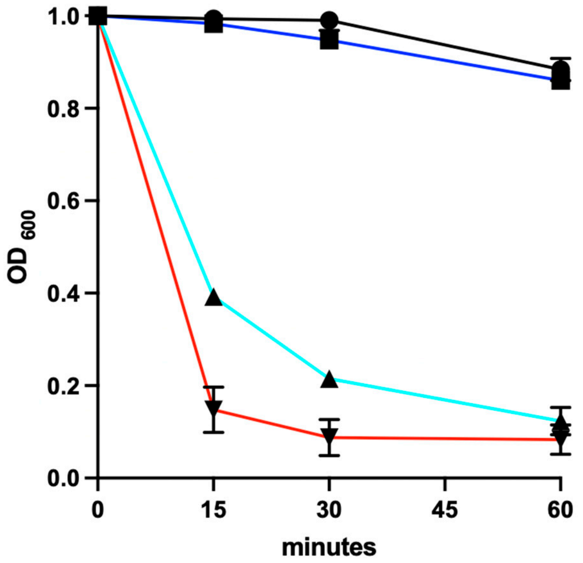


**Figure S6.** The role of germinants on the activity of the EAD. *C. difficile* strain R20291 was grown to mid-log phase in pre-reduced BHI broth. The cells were centrifuged and resuspended in PBS only (black line), PBS and germinants (0.1% Tc and 50mM glycine) (dark blue line), PBS and 60μg/ml

EAD (light blue line) or PBS + germinants and 60µg/ml EAD (red line). The experiment was performed in triplicate and readings were normalized to the initial value.

*7. SEM images of treated C. difficile.*

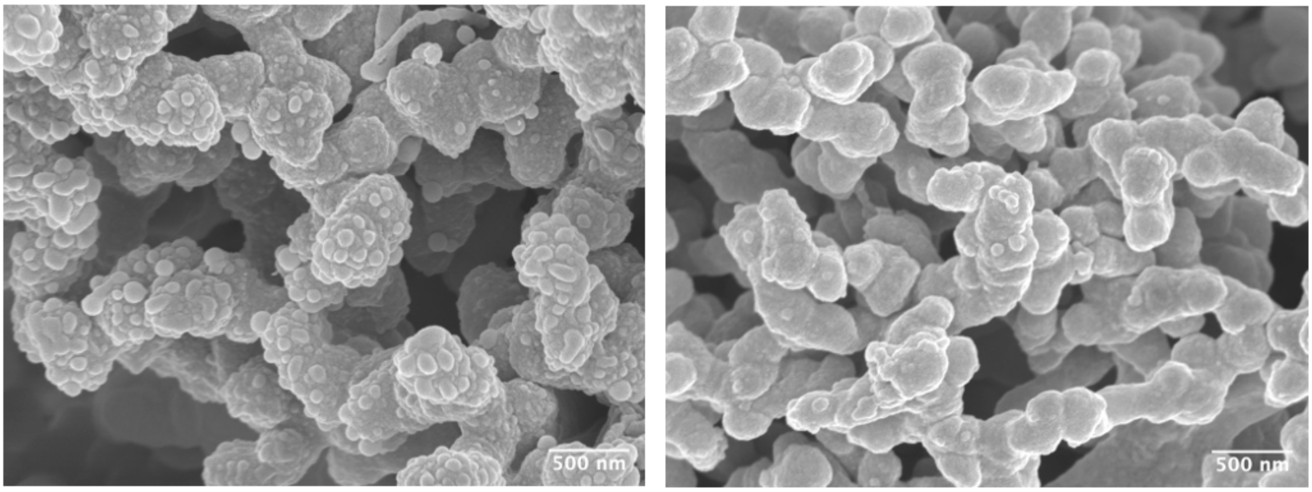


(a) (b)

**Figure S7.** Scanning electron microscope images of *C. difficile* R20291 exposed to the EAD of LysCD6356 and germinants in presence and absence of calcium. Vegetative *C. difficile* strain R20291 was incubated in BHI broth to mid-log phase, after which cells were resuspended in DIW and exposed to 60 ug/ml EAD of LysCD6356 and germinants (0.1% taurocholate and 50 mM glycine) in the presence (b) or absence (a) of 12.5mM calcium chloride for 30 minutes. Images are X 70,000 magnification.

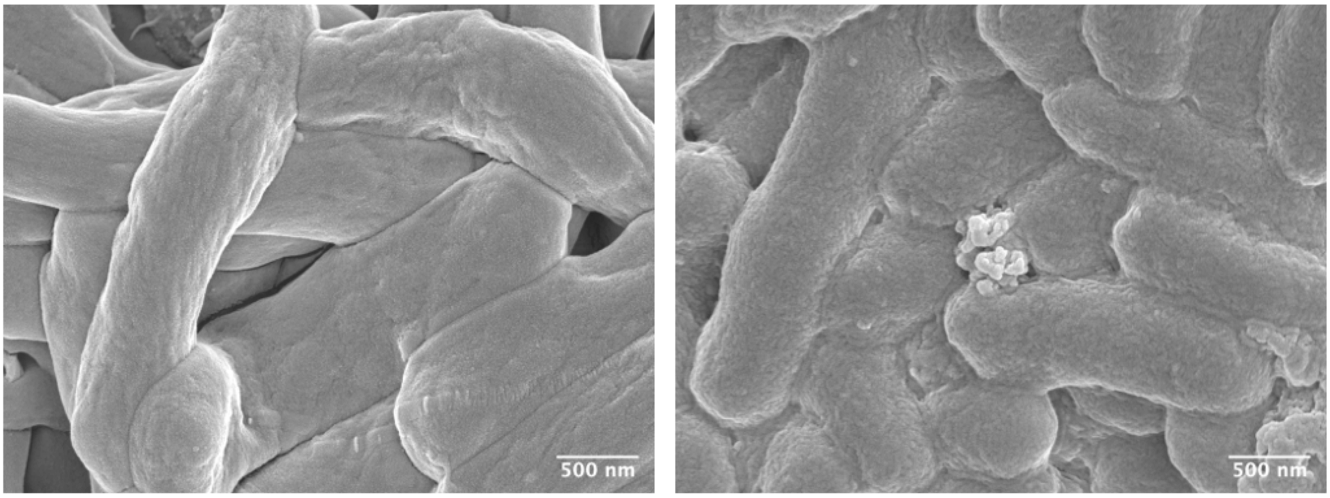


(a) (b)

**Figure S8.** Scanning electron microscope images of *C. difficile* R20291 exposed to germinants in the presence and absence of calcium. Vegetative *C. difficile* strain R20291 was incubated in BHI broth to mid-log phase, after which cells were resuspended in SDW and exposed to germinants (0.1% taurocholate and 50 mM glycine) in the presence (b) or absence (a) of 12.5mM calcium chloride for 30 minutes. Images are X 70,000 magnification.

Investigating the length of each bacterial group (figure S8) demonstrated a reduction in the length of the group exposed to both germinants and $CaCl_2$. The significant reduction in the size (figure S9) explains the reduction in the $OD_{600}$ readings in figure 4.

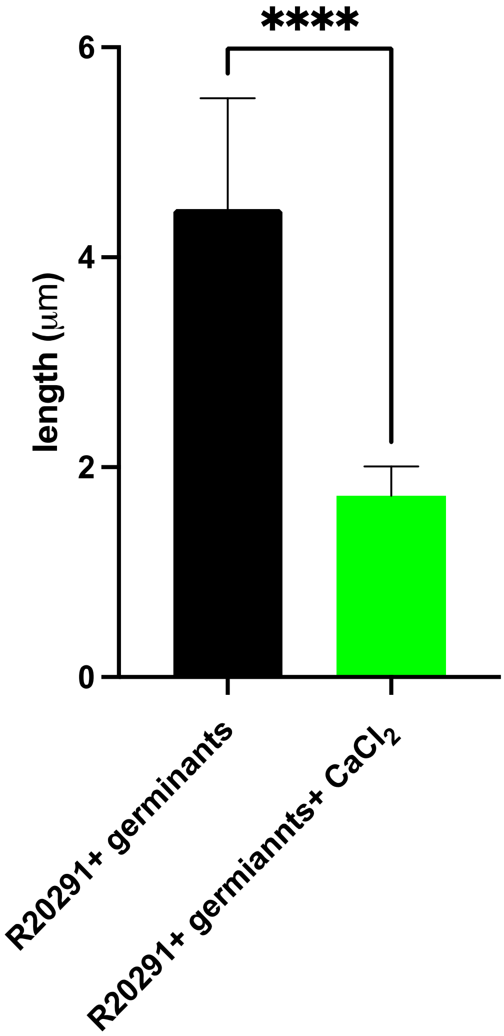


**Figure S9.** Size comparison between vegetative and germinants with and without calcium. Vegetative *C. difficile* strain R20291 was incubated in BHI broth to mid-log phase, after which cells were resuspended in SDW with germinants (0.1% Tc + 50mM glycine) (black bar) or with germinants and $CaCl_2$ (green bar). Cells were then fixed and imaged using SEM. The length of 30 different cells was measured and statistical analysis was performed. $p$ value = < 0.0001 (****).

*8. Phase-Contrast Imaging of spores following germination*

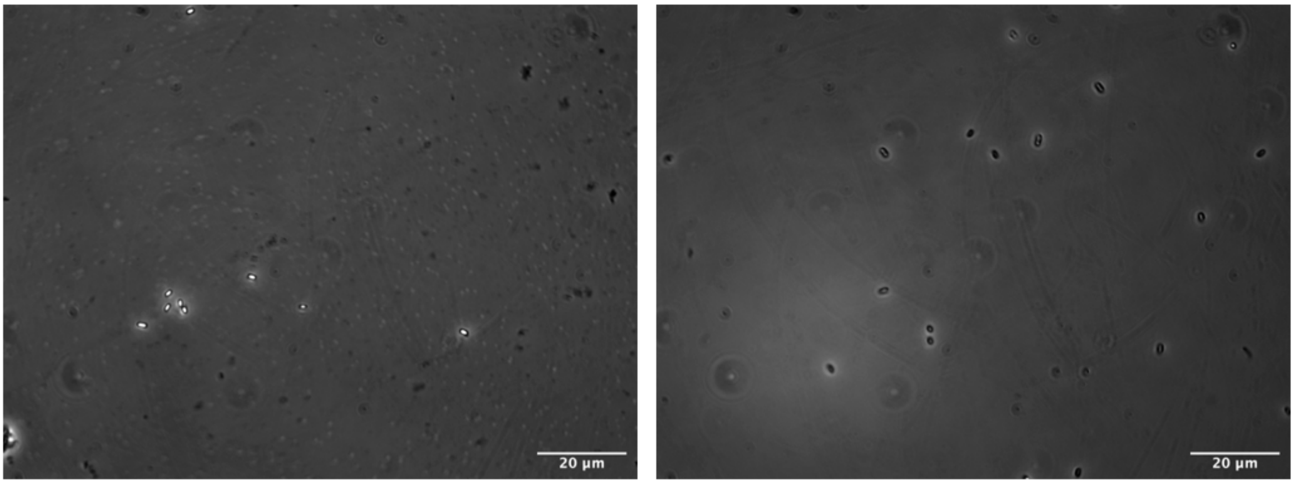


(a) (b)

**Figure S10.** Phase-contrast images of the germination of *C. difficile* spores. Purified spores of *C. difficile* strain R20291 were imaged without germinants (a). Spores appear in phase bright, which indicates ungerminated spores. Following 60 minutes of the germination of *C. difficile* spores with 0.1% sodium taurocholate and 50 mM glycine, the spores appear to form phase dark spores (b). Images were captured using a Leica DMIRB Inverted Leica Modulation Contrast Microscope.

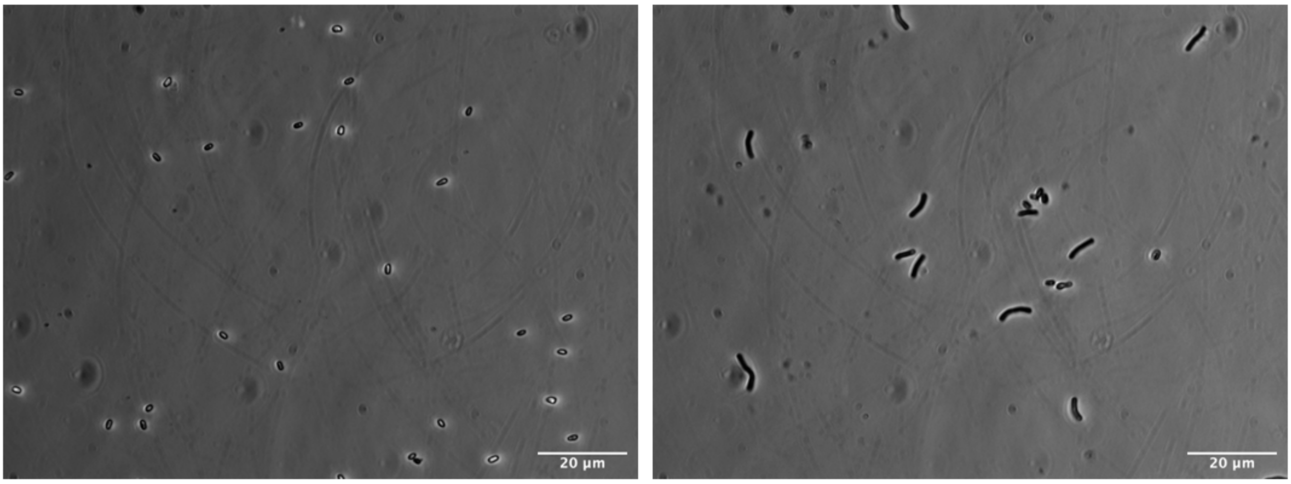


(a) (b)

**Figure S11.** Phase-contrast imaging of *C. difficile* spores R20291 following 150 minutes of germination. Purified *C. difficile* R20291 spores were exposed to 0.1% sodium taurocholate and 50 mM glycine and incubated for 150 minutes at 37o C in SDW (a) or BHI (b). Germinated spores enlarged in size and formed a vegetative form of the bacterium in BHI compared with spores incubated in SDW. Images were captured using a Leica DMIRB Inverted Leica Modulation Contrast Microscope.
